# Enhanced Susceptibility of Galectin-1 Deficient Mice to Experimental Colitis

**DOI:** 10.3389/fimmu.2021.687443

**Published:** 2021-06-28

**Authors:** Raquel Fernandez-Perez, Mercedes Lopez-Santalla, Rebeca Sánchez-Domínguez, Omaira Alberquilla, Irene Gutiérrez-Cañas, Yasmina Juarranz, Juan A. Bueren, Marina I. Garin

**Affiliations:** ^1^ Division of Hematopoietic Innovative Therapies, Centro de Investigaciones Energéticas, Medioambientales y Tecnológicas (CIEMAT) and Centro de Investigación Biomédica en Red de Enfermedades Raras (CIBER-ER), Madrid, Spain; ^2^ Advanced Therapy Unit, Instituto de Investigación Sanitaria Fundación Jiménez Díaz (IIS-FJD/UAM), Madrid, Spain; ^3^ Departamento de Biología Celular, Facultad de Biología y Medicina, Instituto de Investigación Sanitaria Hospital 12 de Octubre (imas12), Universidad Complutense de Madrid, Madrid, Spain

**Keywords:** galectin-1, inflammatory bowel disease, immune regulation, DSS, cell therapy, regulatory T cells

## Abstract

Galectin-1 is a *β*-galactoside-binding lectin, ubiquitously expressed in stromal, epithelial, and different subsets of immune cells. Galectin-1 is the prototype member of the galectin family which shares specificity with *β*-galactoside containing proteins and lipids. Immunomodulatory functions have been ascribed to endogenous galectin-1 due to its induction of T cell apoptosis, inhibitory effects of neutrophils and T cell trafficking. Several studies have demonstrated that administration of recombinant galectin-1 suppressed experimental colitis by modulating adaptive immune responses altering the fate and phenotype of T cells. However, the role of endogenous galectin-1 in intestinal inflammation is poorly defined. In the present study, the well-characterized acute dextran sulfate sodium (DSS)-induced model of ulcerative colitis was used to study the function of endogenous galectin-1 during the development of intestinal inflammation. We found that galectin-1 deficient mice (*Lgals1^−/−^* mice) displayed a more severe intestinal inflammation, characterized by significantly elevated clinical scores, than their wild type counterparts. The mechanisms underlying the enhanced inflammatory response in colitic *Lgals1^−/−^* mice involved an altered Th17/Th1 profile of effector CD4^+^ T cells. Furthermore, increased frequencies of Foxp3^+^CD4^+^ regulatory T cells in colon lamina propria in *Lgals1^−/−^* mice were found. Strikingly, the exacerbated intestinal inflammatory response observed in *Lgals1^−^* ^/^ *^−^* mice was alleviated by adoptive transfer of wild type Foxp3^+^CD4^+^ regulatory T cells at induction of colitis. Altogether, these data highlight the importance of endogenous galectin-1 as a novel determinant in regulating T cell reactivity during the development of intestinal inflammation.

## Introduction

Galectins are among the best-characterized glycan-binding proteins that play key roles in multiple immune cell processes (1). They formed a family of 15 mammalian galactoside-binding proteins that exhibit a consensus amino acid sequence in their carbohydrate recognition domains (CRDs) (2). Among these, galectin-1, which is considered as a prototype member of the galectin family and is a homodimer of 14.5 kDa that plays pivotal roles in modulating acute and chronic inflammatory responses. Galectin-1 is ubiquitously expressed by stromal, epithelial, and endothelial cells from different tissues. Within the immune system myeloid cells like macrophages, as well as subsets of B and T cells including regulatory T cells (Tregs), have been described to express galectin-1 (3–5).

Perillo et al. first demonstrated that expression of galectin-1 in the thymic stroma contributed to deletion of autoreactive immature thymocytes. In addition, recombinant galectin-1 induced apoptosis of activated human T cells and human leukemia T cell lines (6, 7). Since then, galectin-1 has been described as controlling T cell survival by binding with CD45, CD43, CD7, and even with CD69, all well-known glycosylated membrane receptors, triggering their clustering and polarization in microdomains that induced signaling and possibly transcriptional reprogramming that still remained undefined (7–11). In transcriptomic and proteomic analyses, we found that galectin-1 was significantly expressed and secreted by mouse and human activated Treg cells. The *in vitro* studies showed that the immunosuppressive activity of Treg cells was blocked by the use of anti-galectin-1 neutralizing antibodies. In the spleen, although the Treg frequencies in *Lgals1^−^* ^/^ *^−^* mice were similar to wild type mice, their suppressive potential *ex vivo* was compromised (5). Rabinovich and collaborators have extensively demonstrated that galectin-1 can specifically control the proliferation of Th17, Th1, and cytotoxic CD8^+^ effector T cell responses but not on naïve, Th2 or regulatory T cells, thanks to an activation-specific repertoire of glycans expressed in their cell membrane glycoproteins that are essential for galectin-1 specific binding (12, 13).

In addition to this, acute inflammatory responses can be modulated by recombinant galectin-1 through different mechanisms such as inhibition of neutrophil adhesion and replacement (14, 15) and by modulating maturation and tissue migration of monocytes, dendritic cells, and macrophages (16, 17). Effector T cell responses are modulated by galectin-1 through an array of mechanisms including control of T cell activation, differentiation, secretion of cytokines, and induction of apoptosis (6, 18–22). In recent years, the essential role of recombinant galectin-1 in the control of inflammation has been widely demonstrated in experimental studies for treatment of various diseases, including autoimmune encephalomyelitis (12, 13), arthritis (23, 24), hepatitis (25), autoimmune diabetes (26, 27), and ulcerative colitis (28). In addition to this, mice deficient in the *Lgals1* gene displayed increased susceptibility to develop autoimmune-like diseases such as autoimmune encephalomyelitis, arthritis, and lupus (1). Recently, a spontaneous inflammatory process in salivary glands that resembles Sjögren’s syndrome has been described in aged *Lgals1^−^* ^/^ *^−^* mice (29). Nevertheless, pieces of evidence of the regulatory effects of galectin-1 in *in vivo* models of intestinal inflammation are still scarce and the mechanisms underlying the immunosuppressive effects of endogenous galectin-1 have not been fully elucidated (1, 30).

In the present study, the acute dextran sulfate sodium (DSS)-induced model of colitis was used to study the function of endogenous galectin-1 during the development of intestinal inflammation. Mice deficient in the *Lgals1* gene displayed an increase susceptibility to developing colitis. Colitic *Lgals1^−^* ^/^ *^−^* mice exhibited an altered Th17/Th1 profile of effector CD4^+^ T cells and Foxp3^+^CD4^+^ regulatory T cells (Treg cells) in mesenteric lymph nodes (mLNs) and colon lamina propria (cLP) in colitic *Lgals1^−^* ^/^ *^−^* mice. Interestingly, the exacerbated intestinal inflammatory response observed in *Lgals1^−^* ^/^ *^−^* mice was alleviated by adoptive transfer of wild type Foxp3^+^CD4^+^ regulatory T cells at induction of colitis. Altogether, these findings contribute to delineate the mechanisms underlying the immunosuppressive role of endogenous galectin-1 during intestinal inflammation.

## Materials and Methods

### Mice

B6.Cg-*Foxp3^tm2Tch^*/J (*Foxp3^EGFP+^*mice) and B6.Cg-L*gals1^tm1Rob^*/J (*Lgals1^−^* ^/^ *^−^* mice) were purchased from the Jackson laboratory (stock numbers 006772 and 006337, respectively). *Foxp3^EGFP+^* mice were backcrossed for up to seven generations with *Lgals1^−^* ^/^ *^−^* mice to generate *Lgals1^−^* ^/^ *^−^* mice expressing the EGFP gene under the endogenous *Foxp3* transcription factor promoter (*Foxp3^EGFP+^Lgals1^−/−^* mice) on C57BL/6J background. All experiments were performed using these two mouse strains. For regulatory T cell adoptive transfer experiments, C57BL/6J (stock number 000664) and B6.Cg-L*gals1^tm1Rob^*/J were used as donors. All experiments were performed in accordance with the corresponding regulations regarding experimental animal welfare (RD 223/1998 and Directive 2010/63/EU protocols). The experimental protocol was reviewed and approved by the ethics committee for animal research at the CIEMAT and Comunidad de Madrid (based on the RD 53/2013).

### Colitis Induction

Experimental colitis was induced by the oral administration of dextran sulfate sodium (DSS) (36,000–50,000 MW; MP Biochemicals; Irvine, CA, USA) in drinking water for 7 days *ad libitum*. The degree of intestinal inflammation achieved was induced according to the concentration of DSS used (1.25% DSS; w/v). Body weight and health status of mice were monitored daily and the degree of intestinal inflammation induced was scored according to the following scale (1): Body weight loss with respect to day 0 expressed as percentage (0: no loss; 1: 0–5%; 2: 5–10%; 3: 10–20%, 4: >20% loss of weight and 5: no survival) (2); stool consistency (0: normal stools; 1: loose stools; 2: watery diarrhea; 3: watery diarrhea with blood and 4: no survival) and (3) general activity (0: normal; 1–2: moderate activity; 3: null activity and 4: no survival).

### T Cell Isolation and *In Vivo* Transfer

CD4^+^ T cells were isolated from spleens using Mojosort Mouse CD4 T Cell isolation kit according to the manufacturer’s instructions (ref. 480033; BioLegend, San Diego, CA). Regulatory T cells (Treg cells) were enriched from total CD4^+^ T cells by cell sorting based on CD25^high^CD127^low/^ *^−^* antibody expression using a BD Influx Cell Sorter (BD Biosciences). The purity of the sorted T cell populations was above 96%. For Treg cells’ adoptive transfer experiments, one million CD25^high^CD127^low/^ *^−^*CD4^+^ Treg cells isolated from wild type C57BL/6J or B6.Cg-L*gals1^tm1Rob^*/J mice *Lgals1^−/−^* mice were infused intraperitoneally into *Foxp3^EGFP+^Lgals1^−/−^* mice at day 0, following 1.25% DSS administration in drinking water *ad libitum* for seven days.

### Flow Cytometry

Cells from spleen (SPL) and mesenteric lymph nodes (mLNs) were mechanically dissociated with a 70-μm strainer. Red blood cells from SPL were lysed using Red Blood Cell Lysis Buffer-CN (Roche, ref. No. B00003). Colon lamina propria mononuclear cells (cLP) were isolated using Lamina Propria Dissociation Kit (ref. No. 130-097-410, Miltenyi Biotec GmbH, Germany) according to the manufacturer’s instructions. Cell suspensions were stained with specific fluorescently conjugated antibodies listed in **Table 1**. For intracellular analysis of cytokine expression, cells were stimulated with 5 ng/ml phorbol myristate acetate (PMA, Sigma-Aldrich; St Louis, MO, USA), 500 ng/ml ionomycin (Sigma-Aldrich; St Louis, MO, USA), TAPI-1 (ENZO Life Sciences; Lausen, Switzerland; BML-PI134-0001), and GolgiStop (Becton Dickinson; Franklin Lakes, NJ, USA) for 5 h. Intracellular staining was performed using BD Pharmingen Transcription Factor Buffer Set (Ref. 562574, BD Bioscience, Franklin Lakes, NJ, USA). Dead cells were excluded using 4′,6-Diamidino-2-phenylindole (DAPI; Sigma Aldrich; St. Louis, USA; D9542). All samples were acquired by using a BD LSRFortessa cell analyzer (BD Biosciences). Cell samples were acquired using BD FACS DIVA™ software (BD Biosciences). Data analysis was performed using FlowJo™ v10.6.2 software (Tree Star, Ashland, OR, USA).

**Table 1:** Antibodies used for flow cytometry analysis.

Primary mouse Ab	Source	Clone	Fluorophore	Supplier
CD45	Rat	30-F12	PerCP	Miltenyi Biotec
CD4	Rat	GK1.5	PE-Cy7	Biolegend
	Rat	RM4-5	BV711	Biolegend
	Rat	H129.19	FITC	BD Pharmigen
	Rat	H129.19	PE	BD Pharmigen
CD3	Hamster	145-2C11	PE-Vio770	Miltenyi Biotec
CD25	Rat	PC61.5	PE	BD Pharmigen
CD127	Rat	A7R34	APC	eBiocience
Foxp3	Rat	FJK-165	APC	Invitrogen
Ki67	Rat	SolA15	eF450	eBiocience
IL10	Rat	JES516E3	BV711	BD Pharmigen
T-Bet	Mouse	eBio4B10	PE-Cy7	eBiocience
ROR*γ*t	Rat	AFKJS	APC	eBiocience

### Histology

Colon tissues were fixed in formaldehyde for 24 h, washed in phosphate-buffered saline (PBS), and embedded in paraffin. For histological evaluation hematoxylin and eosin (H&E) colon cross-sections were performed in order to analyze tissue structure, cell infiltrations, edema, and general tissue damage.

### Semiquantitative Real-Time PCR

RNA from mLNs and colon biopsies was isolated using TriReagent method (Sigma Aldrich). RNA, 2 µg, was reverse transcribed using a High Capacity cDNA Reverse Transcription Kit (Life Technologies). Relative gene expression was determined by semiquantitative RT-PCR analysis using TaqMan Gene Expression Master Mix (Applied Biosystems, Waltham, MA, USA) (**Table 2**). Each sample was normalized with *β*-actin using the formula 2−ΔCt. Amplification was performed in a 7900HT Fast Real-Time PCR System apparatus (Applied Biosystems).

**Table 2:** Sequences of primers used in RT-PCRs.

Gene	GenBank accession no.	Assay ID (TaqMan^®^)
Actb (*β*-actin)	AK075973.1	Mm01205647_g1
Tbx21 (T-bet)	NM_019507.2	Mm00450960_m1
Rorc (RORϒt)	NM_001293734.1	Mm01261022_m1
Foxp3 (FoxP3)	NM_001199347.1	Mm00475162_m1
Il-10 (IL-10)	NM_010548.2	Mm01288386_m1

### Cytokines in Plasma

Peripheral blood was collected on the day of sacrifice. The samples were centrifuged at 12,000 rpm for 10 min, and plasma was stored at −20°C. Cytokine concentrations were measured using a mouse LEGENDplex multiplex assay (LEGENDplex by Biolegend, 8999 BioLegend Way, San Diego, CA 92121) according to manufacturer’s instructions. Samples were acquired on a BD LSRFortessa cell analyzer (BD Biosciences).

### Statistical Analysis

Results are expressed as mean, and error bars represent standard error of the mean (SEM). Normal distribution was analyzed by Shapiro–Wilk tests. Then, experimental groups were statistically evaluated using the non-parametric unpaired Mann–Whitney U test. ‘P’ values that were considered statistically significant were p <0.05. (*; # p < 0.05, **; ##p < 0.01, ***p < 0.001 and ****p < 0.0001). Survival curves were represented using Kaplan–Meier curves, and differences in experimental group survival were calculated by the long-rank (Mantel–Cox) test. Statistical analysis was performed with GraphPad Prism 9.

## Results

### Absence of Endogenous Galectin-1 Leads to Increased Severity of DSS-Induced Colitis

**Figure 1:**
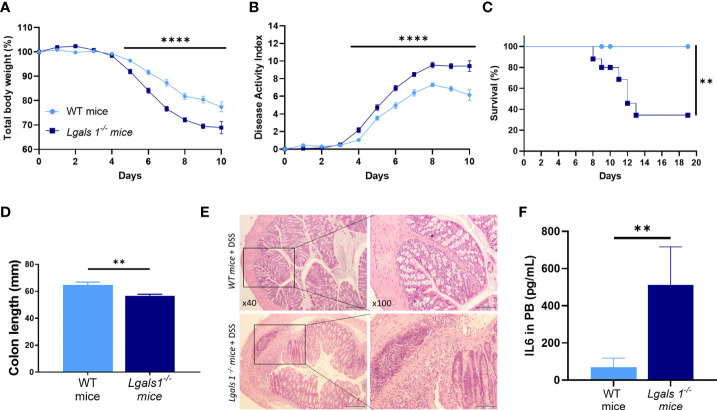
*Lgals1* ^−/−^ mice exhibited enhanced susceptibility to DSS-induced colitis. Colitis was induced with 1.25% DSS w/v in drinking water for 7 days *ad libitum*. Mice were monitored daily. **(A)** Total body weights with respect to the day 0 (%). **(B)** Disease activity index (DAI). **(C)** Survival of mice (n = 23 for WT and n = 25 for *Lgals1^−/−^* mice per group)**. (D)** Colon lengths (n = 18 for WT and n = 17 for *Lgals1^−/−^* mice per group). **(E)** Representative H&E colon tissue (×40 left, ×100 right magnifications, respectively). **(F)** IL-6 plasma concentration (pg/ml) (n = 8 for WT and n = 6 for *Lgals1^−/−^* mice per group). Data are representative of three independent experiments. Statistical analysis was done using Mann–Whitney U test. Data are shown as mean ± SEM, **p < 0.01 and ****p < 0.0001.

To investigate whether endogenous galectin-1 plays a role in experimental colitis, wild type (WT) and *Lgals1^−/−^* mice were treated with DSS in drinking water for 7 days *ad libitum*. A significantly increased severity of DSS-induced colitis was observed in *Lgals1^−/−^* mice as shown by the increased body weight loss and increased disease activity index (DAI) of these mice with respect to WT mice (**Figures 1A, B**). In addition, survival of *Lgals1^−/−^* mice was dramatically reduced as compared to their WT counterparts (**Figure 1C**). Colon length was also significantly reduced in colitic *Lgals1^−/−^* mice with respect to colitic WT mice (**Figure 1D**). These results were further confirmed by histological evaluation of H&E-stained colon cross-sections where a more severe colitis characterized by increased edema, denser transmural leukocyte infiltration, and increased mucosal erosion was observed in DSS-treated *Lgals1^−/−^* mice with respect to colitic WT mice (**Figure 1E**). Plasma levels of interleukin 6 (IL-6) were also significantly increased in colitic *Lgals1^−/−^* mice compared to colitic WT mice (**Figure 1F**). The enhanced susceptibility of *Lgals1^−/−^* mice to developing DSS-induced colitis with respect to WT mice suggests that endogenous galectin-1 modulates intestinal inflammatory responses.

### Increased Plasticity of Th17 Cells Toward Th1 Profile in DSS-Induced Colitic *Lgals1^−/−^* Mice

**Figure 2:**
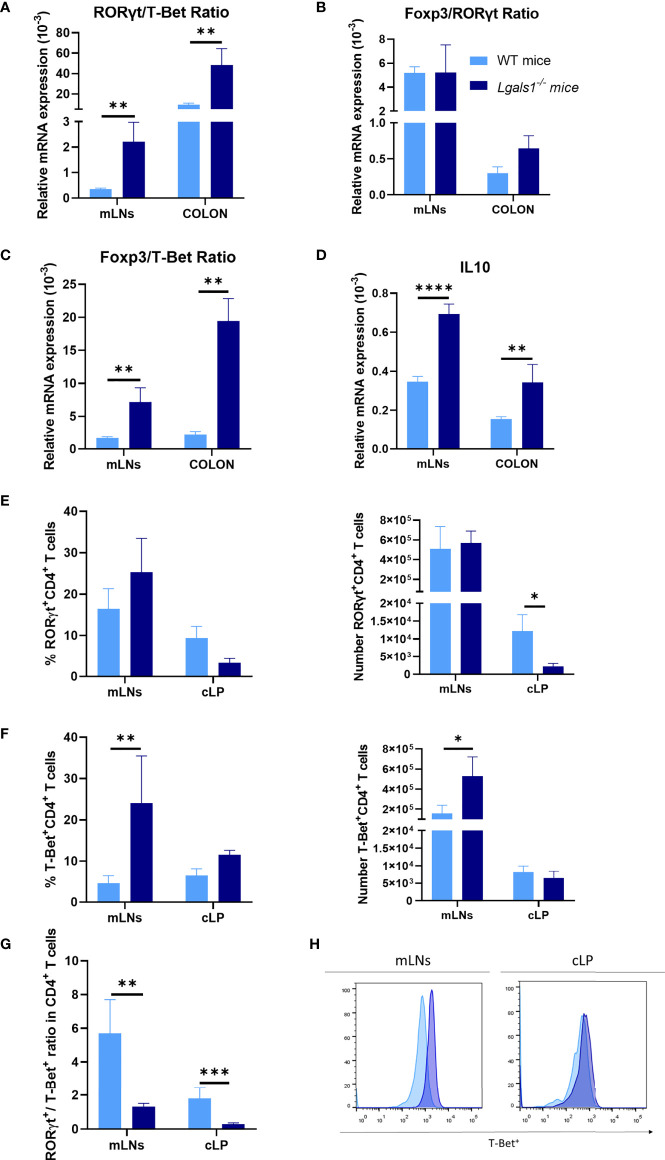
Increased Th1 profile of DSS-induced colitic *Lgals1^−/−^* mice compared to colitic WT mice. mRNA expression of ROR*γ*t, T-Bet, Foxp3, and IL-10 was measured in whole mesenteric lymph nodes (mLNs) and colon biopsies in colitic *wild type* and *Lgals1^−/−^* mice at day 9 (n = 6 for WT and, n = 7 for *Lgals1^−/−^* mice per group). **(A)** ROR*γ*t/T-Bet ratios. **(B)** Foxp3/ROR*γ*t ratios. **(C)** Foxp3/T-Bet ratios. **(D)** Relative mRNA expression of IL-10. Frequencies and absolute numbers of ROR*γ*t^+^CD4^+^ T cells **(E)** and T-Bet^+^CD4^+^ T cells **(F)** measured by flow cytometry. **(G)** ROR*γ*t/T-Bet ratios in CD4^+^ T cells. **(H)** Representative examples of T-Bet^+^ expression by flow cytometry within CD4^+^ of mLNs and cLP in colitic mice (n = 8 for WT and n = 7 for *Lgals1^−/−^* mice per group). Data are representative of two independent experiments. Statistical analysis was done using Mann–Whitney U test. Data are shown as mean ± SEM, *p < 0.05, **p < 0.01, ***p < 0.001 and ****p < 0.0001.

To analyze the Th17/Th1 profile in DSS colitic WT and *Lgals1^−/−^* mice, the expression of mRNA levels of ROR*γ*t and T-Bet transcription factors was determined by quantitative real-time PCR in mLNs and colon. As shown in **Figure 2A**, the ROR*γ*t/T-Bet ratio was significantly increased in colitic *Lgals1* ***^−^*** *^/^* ***^−^*** mice with respect to colitic WT mice in both tissues suggesting that the exacerbated inflammatory response observed in colitic *Lgals1^−/−^* mice is mainly due to an enhanced induction of Th17 cell responses. No differences were determined in the Foxp3/RORγt ratio between colitic WT and *Lgals1* ***^−^*** *^/^* ***^−^*** mice although a tendency to increase in the colon in colitic *Lgals1* ***^−^*** *^/^* ***^−^*** mice was noticed (**Figure 2B**). In contrast, colitic *Lgals1^−/−^* mice had increased Foxp3/T-Bet ratio (**Figure 2C**) together with increased expression of IL10 (**Figure 2D**) both in mLNs and colon. These results indicate that endogenous galectin-1 ameliorates intestinal inflammatory responses mainly by targeting Th17 cell responses in the gut. However, the higher levels of Foxp3 and IL10 mRNAs observed in colitic *Lgals1* ***^−^*** *^/^* ***^−^*** mice were surprising since these results inversely correlated with the exacerbated inflammatory status of the colitic *Lgals1* ***^−^*** *^/^* ***^−^*** mice with respect to colitic WT mice (**Figure 1**).

Effector CD4^+^ T cells responses were further analyzed in colitic mice by flow cytometry. No clear differences were measured in the frequencies and total number of RORγt^+^CD4^+^T cells between colitic WT mice and colitic *Lgals1* ***^−^*** ^/^ ***^−^*** mice in mLNs. However, the absolute number of ROR*γ*t^+^CD4^+^T cells was significantly reduced in cLP in colitic *Lgals1* ***^−^*** ^/^ ***^−^*** mice with respect to the WT colitic mice (**Figure 2E**). On the other hand, a significant increase in the percentage and absolute number of T-Bet^+^CD4^+^T cells was observed in mLNs in colitic *Lgals1* ***^−^*** ^/^ ***^−^*** mice compared to colitic WT mice (**Figures 2F, H**). Colitic WT mice had a significant increase in the ROR*γ*t^+^/T-Bet^+^ balance within CD4^+^ T cells when the absolute number of effector ROR*γ*t^+^CD4^+^T cells and T-Bet^+^CD4^+^T cells in mLNs and cLP was considered (**Figure 2G**). These results may suggest that the exacerbated intestinal inflammatory responses observed in colitic *Lgals1* ***^−^*** ^/^ ***^−^*** mice occurred as a consequence of enhanced plasticity of responder Th17 cells towards a Th1 pathogenic profile in the intestine.

### Increased Frequencies of Foxp3^+^CD4^+^Treg Cells in Colon Lamina Propria in *Lgals1^−/−^* Mice

**Figure 3:**
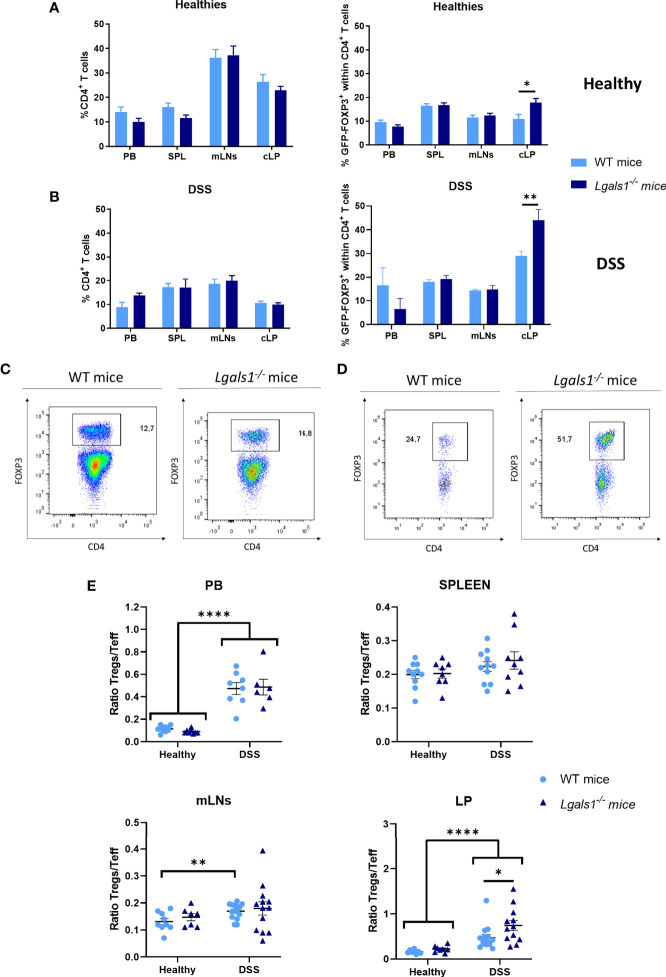
FOXP3^+^CD4^+^ regulatory T cell frequencies were increased in cLP in healthy and colitic *Lgals1^−/−^* mice. **(A)** Frequencies of CD4^+^ T cells and FOXP3^+^CD4^+^ T cells within CD4^+^ T cells in peripheral blood (PB), spleen (SPL), mesenteric lymph nodes (mLNs), and colon lamina propria (cLP) in healthy wild type and *Lgals1^−/−^* mice (n = 10 for WT and n = 8 for *Lgals1^−/−^* mice per group). Data are representative of three independent experiments. **(B)** Frequencies of CD4^+^ T cells and FOXP3^+^CD4^+^ T cells within CD4^+^ T cells in PB, SPL, mLNs, and cLP in DSS colitic wild type and *Lgals1^−/−^* mice (n = 5–15 for WT and, n = 9–13 for *Lgals1^−/−^* mice per group, respectively) at day 9. Data are representative of three independent experiments. A representative example of FOXP3^+^CD4^+^ T cells by dot plots FACS analysis of mLNs **(C)** and cLP **(D)** DSS-treated mice is shown. Numbers represent analyzed population percentages in the respective gates. **(E)** Foxp3^+^CD4^+^ Treg cells (Tregs)/Foxp3^−^CD4^+^ T effector cells (T_eff_) ratios in PB, SPL, mLNs and cLP in healthy and colitic wild type and *Lgals1^−/−^* mice (n = 8–15 for WT and, n = 6–13 for *Lgals1^−/−^* mice per group, respectively). Data are representative of three independent experiments. Statistical analysis was done using Mann–Whitney U test. Data are shown as mean ± SEM, *p < 0.05; **p < 0.01, and ****p < 0.0001.

**Figure 4:**
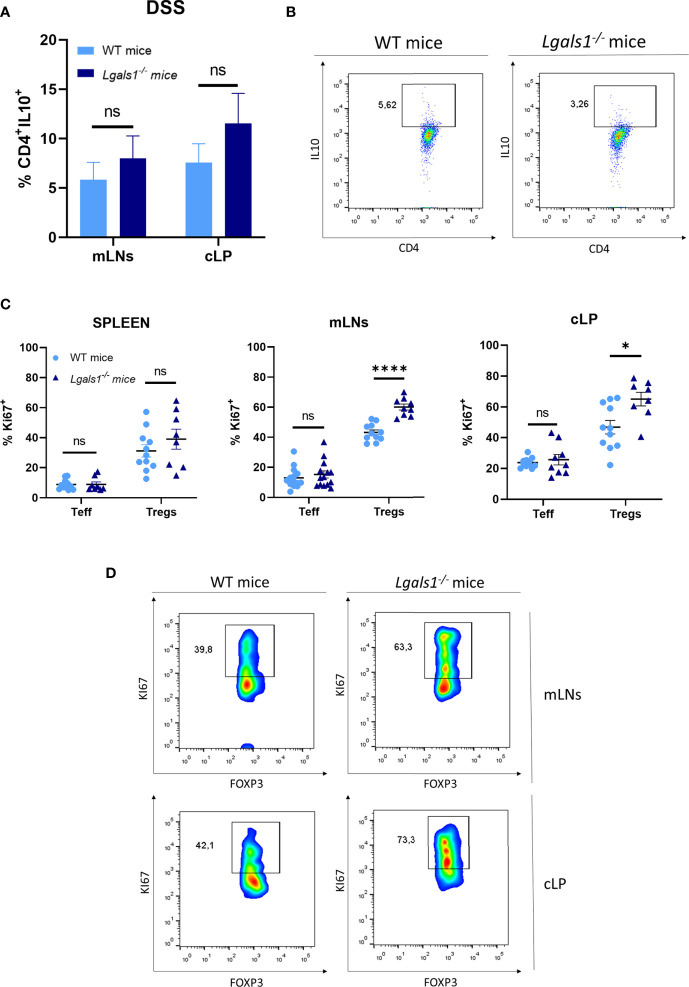
Increased proliferation rate of FOXP3^+^CD4^+^ regulatory T cells in mLNs and cLP of DSS-colitic *Lgals1^−/−^* mice compared to colitic WT mice. **(A)** IL-10^+^CD4^+^ frequencies in mLNs and cLP (n = 7 for WT and, n = 8 for *Lgals1^−/−^* mice per group). Data are representative of two independent experiments. **(B)** Representative examples of CD4^+^IL-10^+^ dot plot FACS analysis of cLP in colitic mice. **(C)** Proliferation rate measured by the expression of Ki67^+^ in T effector cells and FOXP3^+^ Tregs cells in colitic mice (n = 14 for WT and, n = 14 for *Lgals1^−/−^* mice per group). Data are representative of three independent experiments. **(D)** Representative examples of Ki67^+^FOXP3^+^ within CD4^+^ T cells dot plots FACS analysis of mLNs and cLP in colitic mice. Numbers represent analyzed population percentages in the respective gates. Statistical analysis was done using Mann–Whitney test. Data are shown as mean ± SEM, *ns*, not significant; *p < 0.05 and ****p < 0.0001.

We next examined whether DSS-induced gut inflammation altered the frequency of total CD4^+^ T cells as well as Foxp3^+^CD4^+^Treg cells in different tissues. To do this, leukocytes isolated from peripheral blood (PB), spleen, mLNs, and cLP were analyzed by flow cytometry in healthy and DSS-colitic mice. No differences were observed in the frequency of CD4^+^ T cells in WT and *Lgals1* ***^−^*** ^/^ ***^−^*** mice in healthy and DSS inflamed mice. Interestingly, increased frequencies of Foxp3^+^CD4^+^Treg cells were observed in cLP in healthy *Lgals1* ***^−^*** ^/^ ***^−^*** mice with respect to healthy WT mice (**Figures 3A, B**). DSS-induced colitis was associated with increased percentages of Foxp3^+^CD4^+^Treg cells in cLP in both WT mice and *Lgals1* ^−/−^ mice with respect to their control non-inflamed counterparts (**Figures 3A, B** right; **D**). No differences in Foxp3 frecuencias were found in other organs, such as mLNs between both genotypes (**Figure 3C**). Conversely, no significant differences were found in the, frequencies of IL10^+^CD4^+^ T cells in colitic WT and *Lgals1* ***^−^*** ^/^ ***^−^*** mice in mLNs or in cLP (**Figures 4A, B**). The Foxp3^+^CD4^+^Treg cells (Tregs)/Foxp3***^−^***CD4^+^ Teffector cells (Teff) ratio was increased in both WT and *Lgals1* ***^−^*** ^/^ ***^−^*** mice in PB and cLPs as a consequence of DSS-induced intestinal inflammation (**Figure 3E**). These differences were significantly higher in cLP in colitic *Lgals1* ***^−^*** ^/^ ***^−^*** mice with respect to colitic WT mice. mLNs and cLP GFP-Foxp3^+^CD4^+^Treg cells from colitic *Lgals1* ***^−^*** ^/^ ***^−^*** mice exhibited increased proliferative rate as measured by the expression of Ki67 (**Figures 4C, D**). This was in contrast to Teff cells from both colitic WT and *Lgals1* ***^−^*** ^/^ ***^−^*** mice that showed similar proliferation rate (**Figures 4C, D**). Taken together, these data suggest that the intestinal inflammation induced by treatment with DSS promotes Foxp3^+^CD4^+^Treg cell proliferation mainly in mLNs and cLP where the inflammatory insult took place. The proliferative response to the inflammatory challenge was significantly increased in Foxp3^+^CD4^+^Treg cells in colitic *Lgals1* ***^−^*** ^/^ ***^−^*** mice compared to colitic WT Foxp3^+^CD4^+^Treg cells thus leading to an increase in the Tregs/Teff ratio in cLP in colitic *Lgals1* ***^−^*** ^/^ ***^−^*** mice. Despite this, *Lgals1* ***^−^*** ^/^ ***^−^*** mice were unable to mount an acute intestinal inflammatory challenge in the gut (**Figure 1**). These data suggest a reduced *in vivo* functionality of Foxp3^+^CD4^+^Treg cells in colitic *Lgals1* ***^−^*** ^/^ ***^−^*** mice in response to an acute intestinal inflammation challenge.

### Enhanced Conversion of Foxp3^+^CD4^+^ Regulatory T Cells to T-Bet^+^Foxp3^+^CD4^+^Treg Cell in the Absence of Endogenous Galectin-1 During the Development of Colitis

**Figure 5:**
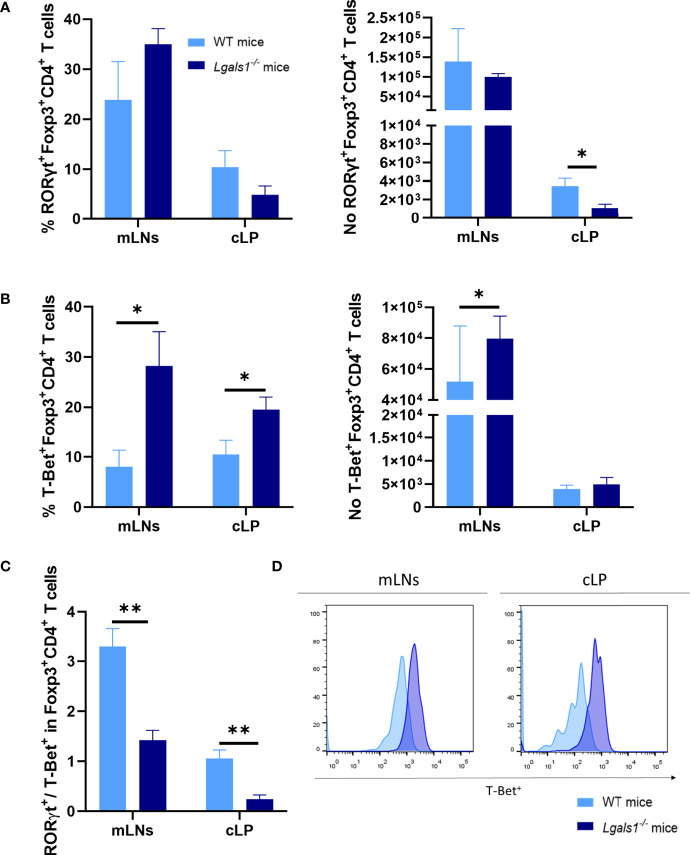
Increase frequencies of T-Bet^+^Foxp3^+^CD4^+^ regulatory T in the absence of endogenous galectin-1 during DSS-induced colitis. Flow cytometry was used to measure ROR*γ*t and T-Bet frequencies in mLNs and cLP within the Foxp3^+^CD4^+^ Treg cells. **(A)** Frequency of ROR*γ*t^+^Foxp3^+^CD4^+^ T cells and absolute numbers of RORγt^+^Foxp3^+^CD4^+^ T cells. **(B)** Frequency of T-Bet^+^Foxp3^+^CD4^+^ T cells and absolute numbers of T-Bet^+^Foxp3^+^CD4^+^ T cells. **(C)** ROR*γ*t/T-Bet ratio in Foxp3^+^ within CD4^+^ T cells. **(D)** Representative histograms of T-Bet^+^ expression in Foxp3^+^CD4^+^ Treg cells by flow cytometry of mLNs and cLP in colitic mice (n = 8 for WT and, n = 6 for *Lgals1^−/−^* mice per group). Data are representative of three independent experiments. Statistical analysis was done using Mann–Whitney U test. Data are shown as mean ± SEM, *p < 0.05 and **p < 0.01.

Numerous studies have demonstrated that the differentiation program of Foxp3^+^CD4^+^Treg cells is not fixed and *in vivo* a fraction of Treg cells can differentiate into Th17- and Th1-like cells during the course of Th17 and Th1 inflammatory responses, respectively. Therefore, the expression of ROR*γ*t and T-Bet were analyzed by flow cytometry within the Foxp3^+^CD4^+^Treg cells in colitic WT and colitic *Lgals1* ***^−^*** ^/^ ***^−^*** mice. No differences were observed in the frequencies of ROR*γ*t^+^Foxp3^+^CD4^+^Treg cells in colitic WT and colitic *Lgals1* ***^−^*** ^/^ ***^−^*** mice. However, the absolute number of ROR*γ*t^+^Foxp3^+^CD4^+^Treg cells in cLP in colitic *Lgals1* ***^−^*** ^/^ ***^−^*** mice was significantly reduced with respect to colitic WT mice (**Figure 5A**). Strikingly, the percentage of T-Bet^+^Foxp3^+^CD4^+^Treg cells were significantly increased in mLNs and cLP in colitic *Lgals1* ***^−^*** ^/^ ***^−^*** mice compared to colitic WT mice (**Figures 5B, D**). As a consequence, the ROR*γ*t/T-Bet ratio within the Treg cells were significantly increased in colitic WT mice with respect to colitic *Lgals1* ^−/−^ mice (**Figure 5C**). These results suggest that an adequate expression of endogenous galectin-1 prevents the conversion of Foxp3^+^CD4^+^Treg cells into T-Bet-expressing Foxp3^+^CD4^+^T cells thus modulating more effectively acute inflammatory responses *in vivo*.

### Adoptive Transfer of Wild Type CD25^high^CD127^low/^ *^−^* Foxp3^+^CD4^+^Treg Cells but Not *Lgals1^−^* ^/^ *^−^* CD25^high^CD127^low/^ *^−^* Foxp3^+^CD4^+^Treg Cells Reduces the Severity of the Disease in DSS-Induced Colitis in *Lgals1^−^* ^/^ *^−^* Mice

**Figure 6:**
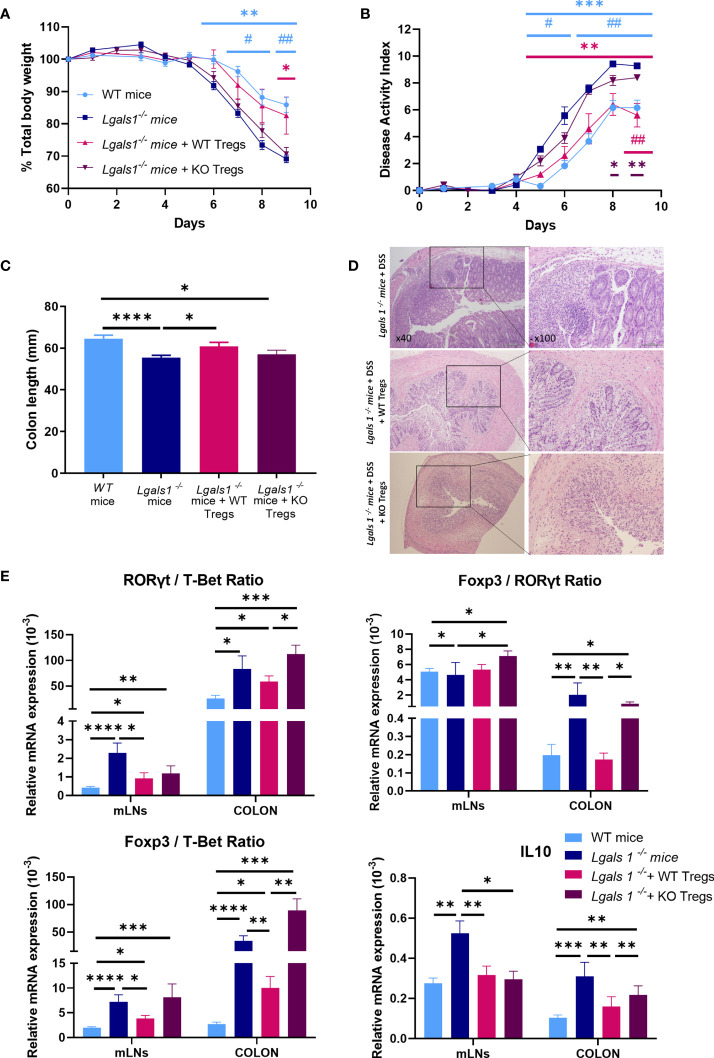
Adoptive transfer of wild type CD25^high^CD127^low/−^Foxp3^+^CD4^+^ Treg cells ameliorated clinical signs of DSS-induced colitis in DSS-treated *Foxp3^EGFP+^Lgals1* ^−/−^ mice. Colitis was induced with 1.25% DSS w/v in drinking water for 7 days *ad libitum* and mice were monitored daily. **(A)** Total body weights. **(B)** Disease activity index (n = 6 for WT, n = 7 *Lgals1^−/−^*, n = 5 for *Lgals1^−/−^* with WT/Tregs and n = 5 for *Lgals1^−/−^* with KO/Tregs mice per group). In statistical analysis, *corresponds to the significant differences with respect to *Lgals1* ^−/−^ mice and # with significant differences with respect to KO/Tregs treated colitic *Lgals1* ^−/−^ mice. **(C)** Colon lengths (n = 24 for WT, n = 25 for *Lgals1^−/−^*, n = 11 for *Lgals1^−/−^* transferred with WT/Tregs and n = 5 for *Lgals1^−/−^* transferred with KO/Tregs mice per group). **(D)** Representative H&E colon tissue of colitic *Lgals1^−/−^* mice, colitic *Lgals1^−/−^* mice infused with 1 × 10^6^ WT/Treg cells and colitic *Lgals1^−/−^* mice infused with 1 × 10^6^ KO/Treg cells respectively (×40 left, ×100 right magnifications). mRNA expression of ROR*γ*t, T-Bet, Foxp3 and IL-10 were measured in mLNs and colon of colitic wild type, *Lgals1^−/−^*, WT/Tregs infused *Lgals1^−/−^* mice and KO/Tregs infused *Lgals1^−/−^* mice (n = 11 for WT, n = 10 for *Lgals1^−/−^*, n = 10 for *Lgals1^−/−^* with WT/Tregs and n = 5 for *Lgals1^−/−^* with KO/Tregs mice per group). **(E)** ROR*γ*t/T-Bet ratio, Foxp3/ROR*γ*t ratio, Foxp3/T-Bet ratio and IL-10 mRNA expression. Data are representative of two independent experiments. Statistical analysis was done using Mann–Whitney U test. Data are shown as mean ± SEM, *p < 0.05; **p < 0.01; ***p < 0.001; ****p < 0.0001; *^#^p >* 0.05, *and ^##^p >* 0.01.

To further define the key role played by Foxp3^+^CD4^+^Treg cells in the exacerbated inflammatory response of *Lgals1* ^−/−^ mice to DSS-induced colitis, we assessed whether adoptive transfer of WT Foxp3^+^CD4^+^Treg cells could reverse the enhanced inflammatory response observed in colitic *Lgals1* ^−/−^ mice. To this end, WT or *Lgals1* ^−/−^ CD25^high^CD127^low/^ ***^−^*** Foxp3^+^CD4^+^Treg cells (WT/Tregs and KO/Tregs, respectively) were isolated by FACs sorting and adoptively transferred into *Foxp3^EGFP+^Lgals1* ***^−^*** *^/^* ***^−^*** mice. As shown in **Figure 6**, WT/Tregs-treated colitic *Lgals1* ^−/−^ mice showed a similar decreased in body weight to colitic WT mice and was significantly reduced with respect to colitic *Lgals1* ^−/−^ mice and KO/Tregs-treated colitic *Lgals1* ^−/−^ mice (**Figure 6A**). The disease activity index of colitic WT and WT/Tregs-treated colitic *Lgals1* ^−/−^ mice were significantly reduced compared to untreated colitic *Lgals1* ^−/−^ mice and KO/Tregs-treated colitic *Lgals1* ^−/−^ mice (**Figure 6B**). Similarly to colitic WT mice, all WT/Tregs and KO/Tregs-treated colitic *Lgals1* ^−/−^ mice survived upon treatment with DSS. The significant differences seen in colon length between colitic WT mice and colitic *Lgals1* ^−/−^ mice were not observed in colitic WT/Tregs treated colitic *Lgals1* ^−/−^ mice. This is in contrast to the colon lengths of KO/Tregs-treated colitic *Lgals1* ^−/−^ mice that were similar to untreated colitic *Lgals1* ^−/−^ mice (**Figure 6C**). Interestingly, WT/Tregs-treated colitic *Lgals1* ***^−^*** ^/^ ***^−^*** mice showed similar histopathology signs of intestinal inflammation to colitic WT mice (**Figure 1E**) with reduced leukocyte infiltration, decreased edema, and mucosal erosion with respect to colitic *Lgals1* ^−/−^ mice. This is in contrast to KO/Tregs-treated colitic *Lgals1* ***^−^*** ^/^ ***^−^*** mice where a reduced leukocyte infiltration with respect to colitic *Lgals1* ***^−^*** ^/^ ***^−^*** mice was noticed (**Figure 6D**).

The expression of mRNA levels of Foxp3, ROR*γ*t and T-Bet transcription factors and IL10 was determined by quantitative real-time PCR in mLNs and colon. In mLNs, the ROR*γ*t/T-Bet ratios were significantly increased in colitic *Lgals1* ***^−^*** ^/^ ***^−^*** mice with respect to colitic WT mice and colitic *Lgals1* ***^−^*** ^/^ ***^−^*** mice treated with either WT/Tregs or KO/Tregs. However, in colon the ROR*γ*t/T-Bet ratios were similar in untreated and WT and KO/Tregs-treated colitic *Lgals1* ***^−^*** ^/^ ***^−^*** mice and significantly higher than in colitic WT mice (**Figure 6E**). Interestingly, the increased Foxp3/T-Bet and Foxp3/ROR*γ*t ratios in mLNs and colon in colitic *Lgals1* ***^−^*** ^/^ ***^−^*** mice were significantly reduced upon adoptive transfer of WT/Tregs in colitic *Lgals1* ***^−^*** ^/^ ***^−^*** mice but not with KO/Treg cells.

In mLNs the high expression levels of IL10 observed in colitic *Lgals1* ^−/−^ mice were significantly reduced upon transfer of WT/Tregs and KO/Tregs and similar to the expression levels in colitic WT mice. In the colons in KO/Tregs-treated colitic *Lgals1* ^−/−^ mice the expression of IL10 was significantly increased with respect to colitic WT mice and WT/Tregs-treated colitic *Lgals1* ^−/−^ mice, whereas no differences were determined when compared to colitic *Lgals1* ^−/−^ mice (**Figure 6E**).

Adoptive transfer of WT/Tregs was sufficient to modulate the enhanced severity of DSS-induced colitis in observed in *Lgals1* ^−/−^ mice in contrast to what was observed in colitic *Lgals1* ^−/−^ mice treated with KO/Treg cells.

## Discussion

The intestine is a distinct microenvironment with a rather unique physiological functions since it is constantly exposed to diverse environmental challenges. Host adaptation in the gut is considered an active process that involves critical interactions between different subsets of immune cells and tissue cells. Foxp3^+^CD4^+^ regulatory T cells are instrumental in enforcing homeostasis at the intestinal barrier surface as illustrated by the development of intestinal inflammation in diseases caused by a primary deficiency in the number or function of Treg cells in the gut (31).

The role of galectin-1 in inflammation has been studied in several *in vitro* and *in vivo* experimental models (23, 32–35). Exogenous administration of galectin-1 affects the viability, proliferation, and Th17 and Th1 responses of non-malignant T cells involved in the progression of cutaneous T-cell lymphoma (36). In homeostasis, *Lgals1* ***^−^*** ^/−^ mice develop normally and do not exhibit any evident alteration compared to galectin-1 sufficient mice (37).

In this study, the acute dextran sulfate sodium (DSS)-induced model of colitis was used to study the function of endogenous galectin-1 during the development of intestinal inflammation. Mice deficient in the *Lgals1* gene displayed an increase susceptibility to developing colitis. We analysed total CD4^+^ T cells and Foxp3^+^CD4^+^ T cells in different tissues in steady state and during DSS-induced acute inflammation. Our data demonstrate that total CD4^+^ T cell infiltration in spleen, mLNs and cLP were similar in both mouse genotypes. Interestingly, in the absence of galectin-1 increased frequencies of Foxp3^+^CD4^+^Treg cells were noticed both in non-inflamed and DSS colitic *Lgals1* ^−/−^ mice compared to WT mice in cLP. Furthermore, no differences were observed in the frequencies of IL10^+^CD4^+^ T cells between both mouse genotypes thus ruling out involvement differences in peripherally induced regulatory CD4^+^ T cells. These results were consistent with a previous study where the frequency of IL10-expressing Treg cells in DSS-treated mice was similar between mouse strains with different susceptibilities to colitis development (38).

The Treg/Teff balance in colitic mice was significantly increased in cLP in colitic *Lgals1* ^−/−^ mice. However, *Lgals1* ^−/−^ mice exhibited an exacerbated inflammatory response to DSS-induced colitis as shown by the enhanced total body losses and disease activity indexes that ultimately led to significantly reduced survival of colitic *Lgals1* ^−/−^ mice compared to WT mice. It is of interest that the proliferative rate of CD4^+^ effector T cells was similar in colitic WT and *Lgals1* ^−/−^ mice as evidenced by Ki67 staining. Conversely, in mLNs and cLP the proliferation rate of Foxp3^+^CD4^+^ Treg cells in colitic *Lgals1* ^−/−^ mice was higher than in colitic WT mice. Our results are in agreement with a previous study conducted in a similar model of acute colitis induced by DSS where the authors demonstrated that Treg cells were required to limit the severity of intestinal inflammation. This was accompanied by an increased number of Treg cells in cLP with higher proliferation rate in both mLNs and in cLP than in effector T cells (39). In IBD patients, increased numbers of Tregs have been found in the inflamed areas of the intestine (40, 41) further confirming that intestinal inflammation is associated with increased frequencies of Treg cells in the inflamed intestine.

ROR*γ*t/T-Bet ratios in mLNs and colon in colitic *Lgals1* ^−/−^ mice were increased with respect to colitic WT mice when assayed by RT-PCR. These results were consistent with studies conducted by Toscano et al. were glycosylation-dependent immune modulatory circuits governed by galectin-1 specifically acted on Th17 and Th1 effector T cells (13). As a consequence, no differences were measured in the Foxp3/ROR*γ*t ratios in mLNs and cLP in both mouse genotypes, mostly due to the increased in the percentages of Foxp3^+^CD4^+^ Treg cells in mLNs and cLP in *Lgals1* ^−/−^ mice. Additionally, relative IL10 mRNA expression levels were higher in colitic *Lgals1* ^−/−^ mice than in colitic WT mice although by flow cytometry the frequencies of IL10^+^CD4^+^ T cells were similar in WT and *Lgals1* ^−/−^ mice. It could be that an exacerbated IL-10 mediated innate immune response is triggered in colitic *Lgals1* ^−/−^ mice to counteract the severe inflammation in the intestine.

ROR*γ*t/T-Bet ratios within the CD4^+^ T cells in mLNs and cLP were significantly reduced in colitic *Lgals1* ^−/−^ mice with respect to colitic WT mice when assayed by flow cytometry. Hence, in the absence of endogenous expression of galectin-1 the increased pathogenic profile of colitic *Lgals1* ^−/−^ mice compared to WT mice directly correlated with increased frequencies and number of T-Bet^+^CD4^+^ T cells in mLNs and cLP leading to a shift in the ROR*γ*t/T-Bet balance in colitic *Lgals1* ^−/−^ mice.

Although considered finally differentiated, numerous studies have shown that Treg cells have certain functional plasticity by acquiring the expression of the master transcription factors that define the T helper cell subsets they are modulating (42, 43). This is a very dynamic process that is also accompanied by coexpression of other master transcription factors (42, 44). Previous studies have demonstrated that expression of T-Bet by Treg cells is required for adequate modulation of Th1 cell responses. Koch et al. first observed that in an inflammatory environment characterized by high levels of IFN*γ* a subset of T-Bet^+^Foxp3^+^ Treg cells emerged. The IFN*γ*-induced T-Bet^+^Foxp3^+^ Treg cells upregulated the expression of CXCR3 allowing the preferential migration of the induced CXCR3^+^T-Bet^+^Foxp3^+^ Treg cells to tissues where a Th1 active response was taking place (44). In our study we show the accumulation of T-Bet^+^Foxp3^+^CD4^+^ Treg cells in mLNs and cLP in colitic *Lgals1* ^−/−^ mice. ROR*γ*t/T-Bet balance within the Foxp3^+^CD4^+^ Treg cells was significantly reduced in colitic *Lgals1* ^−/−^ mice as compared to colitic WT mice. Interestingly, Di Giovangiulio et al. recently demonstrated that Th1-like Treg cells sustained intestinal inflammation since Treg-specific T-Bet conditional ablation knockout mice developed milder DSS-induced colitis than their wild type counterparts. The authors claimed that the T-Bet^+^Foxp3^+^CD4^+^ Treg cells in fact acted as enhancers of Th1 cell responses during the early phases of the inflammation (38). In agreement with this observation, miR146a-deficient IFN*γ* ^+^ Tregs induced increased Th1 effector responses compared to wild type mice (45). Furthermore, in a lethal model of *T. gondii* infection, Oldenhove at. al. demonstrated that T-Bet^+^ IFN*γ* ^+^Foxp3^+^CD4^+^ Treg cells were responsible for the exacerbated Th1 immune response in the infected mice (46). Future studies are warranted aiming at fully defining the functionality of *in vivo* converted T-Bet^+^Foxp3^+^CD4^+^ Treg cells under certain inflammatory conditions in the gut.

There is an increasing body of evidence on the therapeutic potential of adoptively transferring healthy Treg cells into patients with a wide range of conditions, including IBD and other autoimmune diseases, in an attempt to shift the balance of active inflammation toward a more tolerogenic microenvironment. Cell therapy approaches with Treg cells are thought particularly useful in those pathophysiological conditions where a link of intestinal inflammation is associated with a decline in Treg functionality.

Several groups have shown that transfer of Treg cells into mice leads to clinical and histological improvement of colitis (47–51). To further confirm that in the absence of endogenous galectin-1 a functional defect in the Foxp3^+^CD4^+^Treg cells is involved in the severe response to DSS-colitis observed in *Lgals1* ^−/−^ mice, we adoptively transferred CD25^high^CD127^low/^ ***^−^*** Foxp3^+^CD4^+^Treg cells (WT/TRegs) into colitic *Lgals1* ^−/−^ mice. WT/TRegs treated colitic *Lgals1* ^−/−^ mice showed a colitis that is akin to that of colitic WT mice. Interestingly, the Foxp3 mRNA levels in mLNs and cLP in WT/TRegs treated colitic *Lgals1* ^−/−^ mice were lower than in the untreated colitic *Lgals1* ^−/−^ mice. As a consequence, the increased Foxp3/RORγt and Foxp3/T-Bet ratios observed in colitic *Lgals1* ^−/−^ mice also decreased in the colitic *Lgals1* ^−/−^ mice upon infusion of WT/TRegs. Ultimately, the RORγt/T-Bet balance in mLNs was reduced in colitic *Lgals1* ^−/−^ mice treated with WT/TRegs, whereas in cLP no differences were noticed.

Altogether, these data support the immunomodulatory function of endogenous galectin-1 in DSS-induced colitis. Enhanced Th1 effector T cell responses together with increased frequencies of T-Bet^+^Foxp3^+^ Treg cells in colitic *Lgals1* ^−/−^ mice were involved in the pathophysiological severe phenotype of colitic *Lgals1* ^−/−^ mice. Adoptive transfer of wild type CD25^high^CD127^low/−^Foxp3^+^CD4^+^Treg cells efficiently ameliorated the exacerbated inflammatory response of colitic *Lgals1* ^−/−^ mice whereas galectin-1 null Treg cells were unable to ameliorate the exacerbated inflammatory intestinal inflammation in colitic *Lgals1* ^−/−^ mice further supporting the pivotal role played by galectin-1 of CD25^high^CD127^low/−^Foxp3^+^CD4^+^ Treg cells in modulating the severity of DSS-induced colitis.

Although the precise mechanisms by which mature Tregs utilize galectin-1 during DSS inflammatory challenge are unclear, these data highlight the importance of endogenous galectin-1 as a novel determinant in regulating T cell reactivity during the development of intestinal inflammation. Further studies are warranted to gain more comprehensive understandings in the biological function of galectin-1 in the development, fate and stability of Treg cells in the intestine.

## Data Availability Statement

The raw data supporting the conclusions of this article will be made available by the authors without undue reservation.

## Ethics Statement

The animal study was reviewed and approved by Ethics committee for animal research at the CIEMAT and Comunidad de Madrid.

## Author Contributions

RF-P and MG planned the project. RF-P, ML-S, RS-D, OA, IG-C, YJ, and MG performed and analyzed the experiments. RF-P, ML-S, RS-D, OA, IG-C, YJ, JB, and MG wrote and edited the manuscript. All authors contributed to the article and approved the submitted version.

## Funding

This work was supported by Instituto de Salud Carlos III grants (PI17/00027, PI20/00078, RD16/0012/0008, PIE15/00048 and PI17/01161), and cofunded by the European Regional Development Fund (ERDF).

## Conflict of Interest

The authors declare that the research was conducted in the absence of any commercial or financial relationships that could be construed as a potential conflict of interest.
